# PIDD interaction with KEAP1 as a new mutation-independent mechanism to promote NRF2 stabilization and chemoresistance in NSCLC

**DOI:** 10.1038/s41598-019-48763-4

**Published:** 2019-08-27

**Authors:** Lili Ji, Rui Zhang, Jie Chen, Qun Xue, Nadeem Moghal, Ming-Sound Tsao

**Affiliations:** 1 Department of Pathology, Medical College of Nantong University, Nantong, Jiangsu 226001 China; 2 Princess Margaret Cancer Centre, University Health Network, Toronto, Ontario M5G 1L7 Canada; 3 Department of Medical Biophysics, University of Toronto, Toronto, Ontario M5G 1L7 Canada; 4 Department of Tuberculosis, the Sixth Hospital of Nantong, Nantong, Jiangsu 226000 China; 5 Department of Oncology, Jiangyin People’s Hospital, Jiangyin, China; 6 Department of Thoracic Surgery, Affiliated Hospital of Nantong University, Nantong, 226001 Jiangsu China

**Keywords:** Non-small-cell lung cancer, Stress signalling, Non-small-cell lung cancer, Preclinical research, Chemotherapy

## Abstract

Chemotherapy resistance is a major problem in non-small cell lung cancer (NSCLC) treatment. A major mechanism of chemoresistance involves stabilization of the NRF2 transcription factor. NRF2 levels are normally tightly regulated through interaction with KEAP1, an adaptor that targets NRF2 to the CUL3 E3 ubiquitin ligase for proteolysis. In NSCLC, aberrant NRF2 stabilization is best understood through mutations in *NRF2*, *KEAP1*, or *CUL3* that disrupt their interaction. Biochemical studies, however, have revealed that NRF2 can also be stabilized through expression of KEAP1-interacting proteins that competitively sequester KEAP1 away from NRF2. Here, we have identified PIDD, as a novel KEAP1-interactor in NSCLC that regulates NRF2. We show that this interaction allows PIDD to reduce NRF2 ubiquitination and increase its stability. We also demonstrate that PIDD promotes chemoresistance in NSCLC cells both *in vitro* and *in vivo*, and that this effect is dependent on NRF2. Finally, we report that NRF2 protein expression in a NSCLC cohort exceeds the typical incidence of combined *NRF2*, *KEAP1*, and *CUL3* mutations, and that NRF2 expression in this cohort is correlated with PIDD levels. Our data identify PIDD as a new NRF2 regulator, and suggest that variations in PIDD levels contribute to differential chemosensitivities among NSCLC patients.

## Introduction

Lung cancer is the leading cause of cancer-related death worldwide, with the majority of patients having non-small cell lung cancer (NSCLC)^1^. Fifty-seven percent of lung cancer patients are diagnosed with distant metastasis and have a 5-year survival rate of only 4.7%^2^. One of the main obstacles to treatment of NSCLC patients is the common development of resistance to anticancer drugs including platinum-based compounds. Cisplatin [cis-diamminedichloroplatinum(II)] is one of the most widely employed chemotherapies in oncology. Cisplatin and platinum-based analogs are currently used to treat many malignancies, including lung, ovarian, head and neck, bladder, and testicular cancer^3^. However, toxicity and resistance have become major barriers limiting the use and efficacy of platinum-based agents. Multiple mechanisms have been shown to be involved in the protection of cancer cells against anticancer drugs, including phase II detoxifying enzyme genes, antioxidant genes, and drug efflux protein genes^4–7^.

NF-E2-related factor 2 (NRF2) is a transcription factor whose activation in cancer cells has been implicated in resistance to chemotherapy^8^. It directly regulates a battery of downstream anti-oxidant genes, including (1) intracellular redox-balancing proteins (glutamate cysteine ligase, *GCL*; heme oxygenase-1, *HO-1*), (2) xenobiotic metabolizing enzymes (NAD[P]H quinone oxidoreductase-1, *NQO1*), and (3) transporters (multidrug resistance-associated proteins, MRPs). Collectively, these genes function in a vast array of processes to protect against oxidative stress and harmful environmental toxicants and carcinogens. Consistent with these molecular functions, high levels of NRF2 in NSCLC are associated with resistance to chemotherapy and poor prognosis^9^. In functional studies, the loss of Kelch-like ECH-associated protein 1 (KEAP1), a negative regulator of NRF2, increases nuclear accumulation and activation of NRF2 in NSCLC cell lines, and promotes growth and chemoresistance^10^. In addition, silencing of NRF2 through RNAi increases sensitivity to cisplatin in NSCLC cells^11–13^. Thus, the development of therapeutic strategies that effectively interfere with NRF2 activity offer great promise for overcoming chemoresistance.

NRF2 activity is highly regulated. It shifts from essentially non-existent under healthy conditions to highly active during stress. This regulation primarily occurs through modulation of NRF2 proteolysis. Under non-stressed conditions, NRF2 is constitutively degraded through interaction with its binding partner KEAP1, which recruits the CUL3-E3 ubiquitin ligase to stimulate proteasomal degradation of NRF2^14^. Upon exposure to oxidative stress or chemopreventive compounds like sulforaphane and curcumin, modification of the cysteine residues on KEAP1 imposes a conformational change that disrupts the binding between KEAP1 and NRF2, resulting in diminished NRF2 ubiquitination and increased NRF2 protein levels and target gene expression^15,16^. The critical importance of this form of regulation to NRF2 activity is supported by the discovery of gain-of-function mutations in *NRF2* that disrupt regulation by KEAP1-CUL3, and loss-of-function mutations in *KEAP1* and *CUL3* in several cancer types^17^. A growing body of evidence suggests that KEAP1 could be a focal point for additional mechanisms of NRF2 regulation. In tumors, NRF2 levels are sometimes increased in the absence of genomic alterations in the *NRF2*, *KEAP1*, and *CUL3* genes. Several studies suggest that this might occur through elevated expression of proteins that compete with NRF2 for binding to KEAP1, and thus, sequester KEAP1 away from NRF2, preventing its ubiquitination. This list of proteins currently includes p62 (SQSTM1), WTX, PALB2 and DPP3^18–21^.

To study potential regulation of NRF2 activity by KEAP1-interacting proteins in NSCLC, we used KEAP1 immunoprecipitation-mass spectrometry (IP-MS) to identify KEAP1-interactors in a NSCLC cell line. Using this approach, we identified P53-induced protein with a death domain (PIDD) as a novel binding partner for KEAP1. We also provide evidence for PIDD being a new clinically relevant regulator of NRF2 and NSCLC malignancy and chemoresistance, and suggest that its further study may yield insight into novel treatment options for NSCLC.

## Results

### NRF2 regulatory pathway alterations in NSCLC and other cancers

To better understand the extent of involvement of NRF2 activation to chemotherapy resistance in NSCLC, we surveyed NRF2 pathway genomic alterations in NSCLC relative to other major cancers. Using the comprehensive TCGA data available through the cBioportal database (www.cBioportal.org)^22^, we examined DNA amplifications, deletions, and mutations in *NRF2*, *KEAP1*, and *CUL3*. When considering the cumulative alteration frequencies of all three genes as an overall assessment of pathway activation, the top two cancers of 31 surveyed malignancies were lung squamous cell carcinoma (35.96%) and lung adenocarcinoma (23.48%), the two main types of NSCLC, which are shown in Supplementary Fig. S1 (Panel A). The detailed alteration frequencies for individual genes are presented in Supplementary Fig. S1 (Panel B–D) and Supplementary Table S1. These data support an especially prominent role for NRF2 pathway activation in NSCLC and suggest that additional mechanisms may promote NRF2 activation in cases where DNA alterations were not detected.

### PIDD interacts with KEAP1

**Figure 1:**
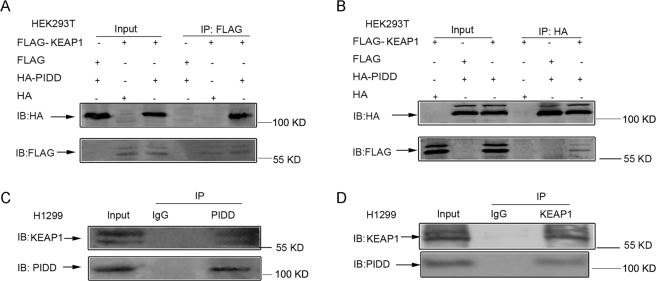
PIDD interacts with KEAP1. (**A,B**) HEK293T cells were transfected with FLAG-KEAP1 and HA-PIDD as indicated. Proteins in total cellular lysates and immunoprecipitations (IP) were analyzed by immunoblotting (IB). (**C,D**) Reciprocal immunoprecipitation of endogenous PIDD and KEAP1 from human H1299 NSCLC cell lysates. (**A–D**) The upper and lower panels were from the same gel. The gel was transferred to the same membrane, which was cut to probe with different antibodies. The entire image of each exposed membrane is shown.

To investigate other potential mechanisms for NRF2 activation, we focused on characterizing novel KEAP1-interacting proteins that might compete with NRF2, and hence, indirectly promote NRF2 stabilization. In pilot discovery experiments, we used immunoprecipitation-mass spectrometry (IP-MS) and H1299 NSCLC cells, which have wild-type *KEAP1* (determined from publicly available data from the Cancer Cell Line Encyclopedia), to identify 58 putative KEAP1-interactors, including P53-induced protein with a death domain (PIDD). We decided to focus on PIDD since it, like NRF2, has been implicated in chemoresistance^23^, which suggested a possible functional connection to NRF2 regulation. To validate the interaction of KEAP1 with PIDD, we first used epitope-tagged constructs and anti-tag antibodies to look for co-immunoprecipitation in transiently transfected HEK293T cells. After transfection of HEK293T cells with FLAG-KEAP1 and HA-PIDD, HA-PIDD was found in anti-FLAG-KEAP1 immunoprecipitates (Fig. 1A), which migrated in the range of ~100 kDa, as detected with the anti-HA antibody. Conversely, FLAG-KEAP1 was found in the reciprocal anti-HA-PIDD immunoprecipitates (Fig. 1B), which consistently migrated as a ~55 kDa doublet, as detected with the anti-FLAG antibody. The Western bands of both PIDD and KEAP1 were consistent with previous papers^24–26^. To determine if the KEAP1 and PIDD interaction could also be detected among endogenously expressed proteins in NSCLC, we also performed co-immunoprecipitation using antibodies directed towards the native proteins. Consistent with the transfection studies, endogenous KEAP1 and PIDD could be co-immunoprecipitated from H1299 NSCLC cells (Fig. 1C,D).

### PIDD reduces the amount of ubiquitinated NRF2

**Figure 2:**
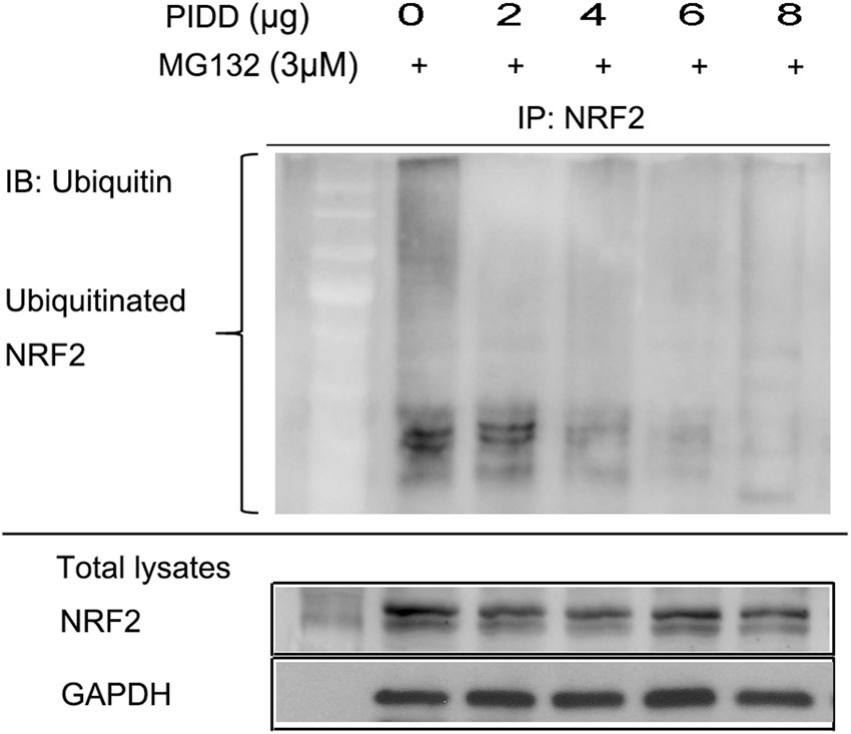
PIDD decreases the amount of ubiquitinated NRF2. HEK293T cells were transfected with different amounts of PIDD expression plasmid in the presence of 10 µM MG132, which blocks degradation of ubiquitinated proteins. NRF2 was then immunoprecipitated (IP) from cell lysates and its level of ubiquitination was quantified by anti-ubiquitin immunoblotting (IB). The upper and lower panels were from the same gel. The gel was transferred to the same membrane, which was cut to probe with different antibodies. The entire image of each exposed membrane is shown.

Since some KEAP1-interacting proteins can indirectly regulate NRF2 levels by sequestering KEAP1and the CUL3-dependent ubiquitylating machinery away from NRF2^26–28^, we next investigated whether PIDD could also regulate NRF2 ubiquitination. HEK293T cells were transfected with increasing doses of a PIDD expression vector and treated with a proteasome inhibitor, MG132, for 4 hours to prevent ubiquitinated NRF2 from being degraded. Endogenous NRF2 was then immunoprecipitated from the lysates and blotted with an anti-ubiquitin antibody. Consistent with our hypothesis, as PIDD levels increased, NRF2 ubiquitination decreased, indicating that PIDD can regulate NRF2 ubiquitination (Fig. 2).

### PIDD promotes chemoresistance in NSCLC cells both *in vitro* and *in vivo*

**Figure 3:**
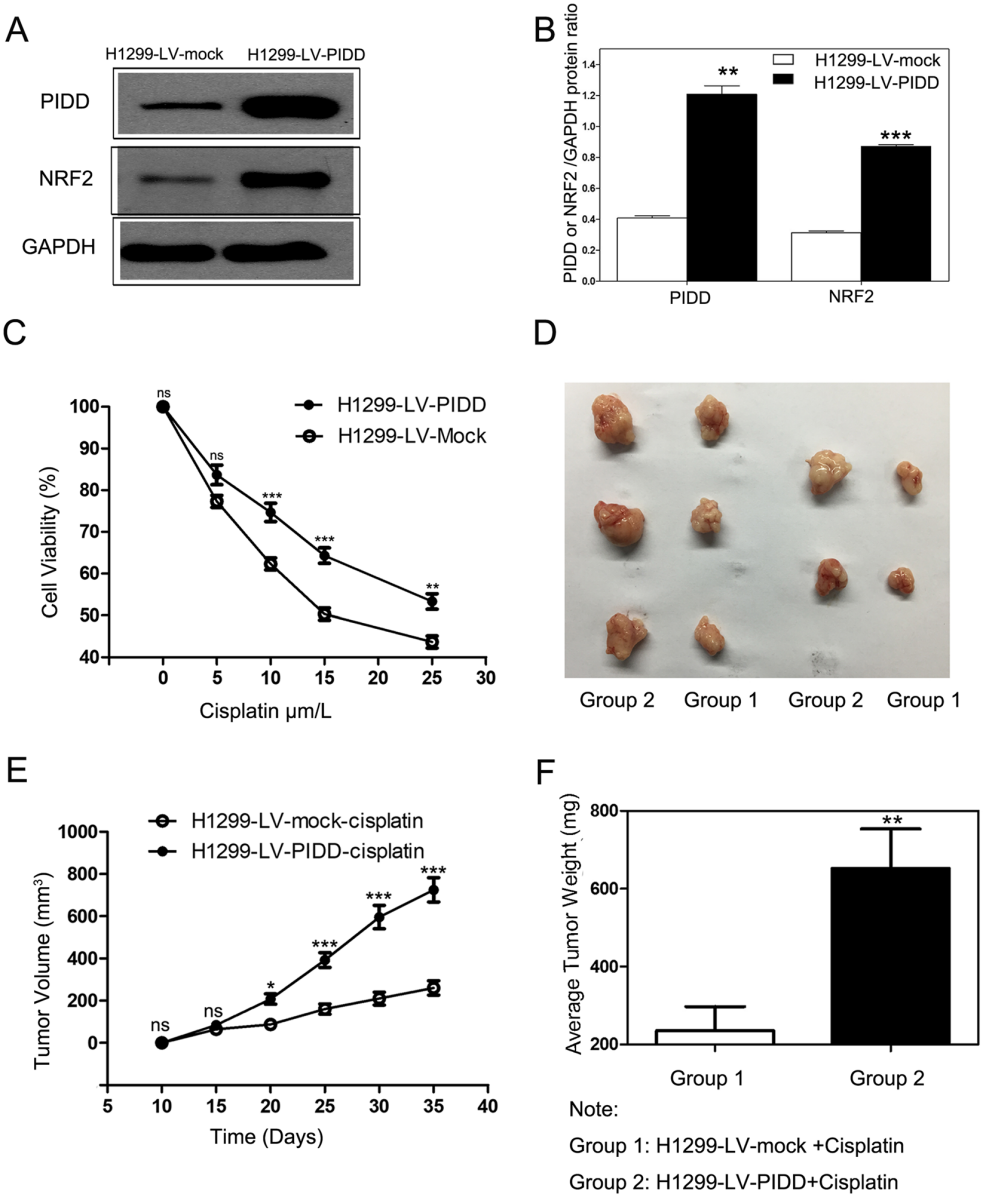
PIDD promotes chemoresistance in NSCLC cells both *in vitro* and *in vivo*. H1299 cells were either infected with a PIDD overexpressing lentivirus (LV-PIDD) or were mock infected (LV-mock). (**A**) Western blotting showed increased levels of PIDD and NRF2 in LV-PIDD-infected cells as compared to LV-mock cells. The upper and lower panels were from the same gel. The gel was transferred to the same membrane, which was cut to probe with different antibodies. The entire image of each exposed membrane is shown. (**B**) Quantification of higher levels of PIDD and NRF2 in lentivirus infected H1299 cells. Data are presented as the densitometric means of three immunoblotting replicates and are normalized to expression of GAPDH. Error bars represent standard error of the mean (SEM) (Two tailed Student’s t-test, **P = 0.0011; ***P < 0.0001). (**C**) LV-PIDD infected H1299 cells showed increased viability as compared to the LV-mock H1299 cells after treatment with different doses of cisplatin for 24 hours. Viability was quantified by the MTT assay. Data are mean ± SEM for three experiments (Two way ANOVA; **P < 0.01; ***P < 0.001; ns, P > 0.05). (**D–F**) Cisplatin sensitivity of LV-mock and LV-PIDD-infected H1299 tumors grown as xenografts *in vivo*. (**D**) Dissected tumors from the two experimental groups showed smaller size of tumors formed by LV-mock H1299 cells treated with cisplatin (Group 1), as compared to tumors formed by LV-PIDD-H1299 cells (Group 2) treated with cisplatin. (**E**) Quantification of tumor volume. The volume of LV-PIDD tumors were significantly larger than the mock group. Data are mean ± SEM for 5 mice per arm (Two way ANOVA; *P < 0.05; ***P < 0.001; ns, P > 0.05). (**F**) Average tumor weight at termination of the experiments. The weights of the LV-PIDD tumors (Group 2) were significantly larger than the mock group (Group 1). The data are means ± SEM reflecting 5 mice per arm. (Two tailed Student’s t-test; **P = 0.0081) compared with the control.

To determine whether PIDD might promote chemoresistance in NSCLC, we used a lentivirus vector to raise PIDD expression in H1299 cells where we found PIDD interacts with KEAP1. In LV-PIDD-infected cells, PIDD expression was increased ~3-fold, which was sufficient to cause a more than 2-fold increase in NRF2 levels, and is consistent with PIDD’s ability to reduce NRF2 ubiquitination (Fig. 3A,B). These changes in PIDD and NRF2 expression in the LV-PIDD-infected cells translated to increased resistance to cisplatin (p < 0.05, Fig. 3C).

To determine whether the increased resistance to cisplatin is also manifested *in vivo*, we generated LV-PIDD H1299 xenografts. Equivalent numbers of LV-PIDD-infected and LV-mock-infected H1299 cells were injected into nude mice and after 10 days, cisplatin treatment was initiated. At the endpoint of the therapeutic regimen, the sizes, volumes, and weights of the LV-PIDD tumors were all significantly larger than the mock group (p < 0.05, Fig. 3D–F), demonstrating increased resistance to cisplatin upon elevation of PIDD levels.

### PIDD-mediated chemoresistance is dependent on NRF2

**Figure 4:**
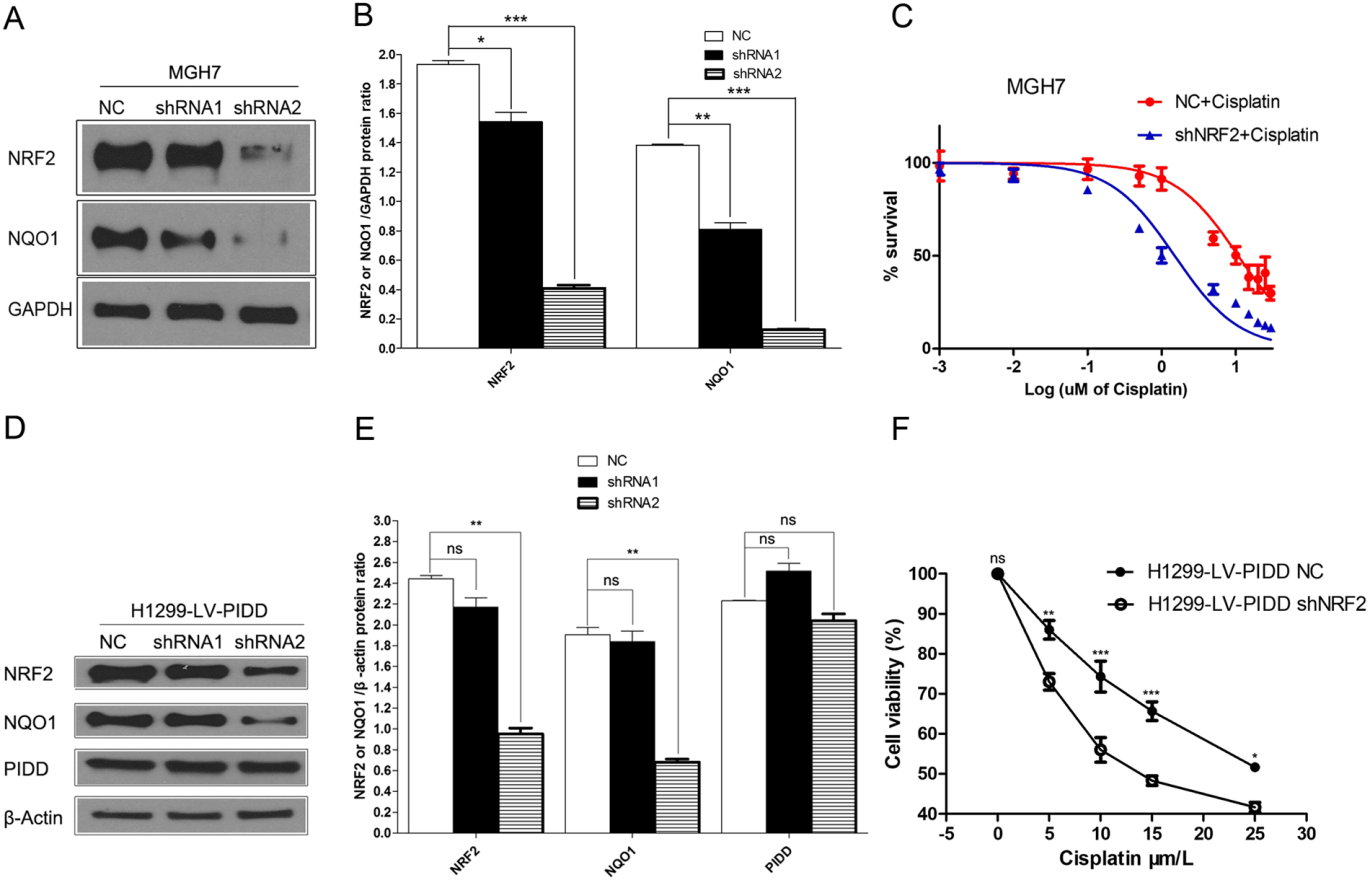
PIDD-induced chemoresistance in NSCLC depends on NRF2. MGH7 or H1299-LV-PIDD cells were infected with shRNA lentiviruses targeting *NRF2* (NRF2-shRNA1 and NRF2-shRNA2) or a non-targeting lacZ shRNA control (NC). (**A**) Western blotting was performed to determine the expression levels of NRF2 and NQO1 in both NC and NRF2-shRNA infected cells. The upper and lower panels were from the same gel. The gel was transferred to the same membrane, which was cut to probe with different antibodies. The entire image of each exposed membrane is shown. (**B**) Quantification of NRF2 and NQO1 protein levels in lentivirus infected MGH7 cells. Data are presented as the densitometric means of three immunoblotting replicates and are normalized to expression of GAPDH. Error bars represent standard error of the mean (SEM) (Two tailed Student’s t-test, *P = 0.0302; **P = 0.0055; ***P < 0.0001). (**C**) Viability of MGH7 cells infected with lentiviruses expressing either NC or shNRF2 (NRF2-shRNA2) after treatment with different doses of cisplatin for 72 hours. Viability was quantified by the MTT assay. (**D**) Western blotting was performed to determine the expression levels of NRF2, NQO1 and PIDD in both NC and NRF2-shRNA infected cells. The upper and lower panels were from the same gel. The gel was transferred to the same membrane, which was cut to probe with different antibodies. The entire image of each exposed membrane is shown. (**E**) Quantification of NRF2, NQO1 and PIDD protein levels in lentivirus infected H1299-LV-PIDD cells. Data are presented as the densitometric means of three immunoblotting replicates and are normalized to expression of ß-actin. Error bars represent standard error of the mean (SEM) (Two tailed Student’s t-test, **P = 0.0019 for NRF2 in shRNA2; **P = 0.0037 for NQO1 in shRNA2). (**F**) NRF2 shRNA2-infected H1299-LV-PIDD cells showed decreased viability as compared to the negative control cells after treatment with different doses of cisplatin for 24 hours. Viability was quantified by the MTT assay. Data are mean ± SEM for three experiments (Two way ANOVA; *P < 0.05; **P < 0.01; ***P < 0.001; ns, P > 0.05).

To explore the mechanism of PIDD-induced chemoresistance in NSCLC, we focused on NRF2, whose stabilization is promoted by PIDD. Using control MGH7 cells, a lung squamous cell carcinoma cell line with high levels of NRF2^29,30^ (Fig. 4A), we identified a lentiviral shRNA construct (shRNA2) that was especially effective at reducing protein levels of NRF2 and the NRF2 target gene, *NQO1* (Fig. 4A,B). MGH7 cells stably infected with NRF2 shRNA2 also had a lower IC50 for cisplatin (1.503 uM vs 10.84 uM for control shlacz infected cells) (Fig. 4C), supporting NRF2 promoting chemoresistance in these cells.

We next used the NRF2 shRNA2 construct to determine if the elevated NRF2 expresssion in the PIDD-overexpressing H1299 cells (H1299-LV-PIDD) contributes to their increased chemoresistance. As observed in MGH7 cells, NRF2 shRNA2 reduced NRF2 and NQO1 protein levels in H1299-LV-PIDD cells, and importantly, without affecting PIDD expression (Fig. 4D,E). The reduction in NRF2 expression also increased sensitivity to cisplatin (Fig. 4F), supporting the PIDD-mediated increase in NRF2 levels driving increased chemoresistance.

### Clinical relevance of PIDD and NRF2 expression in NSCLC

**Figure 5:**
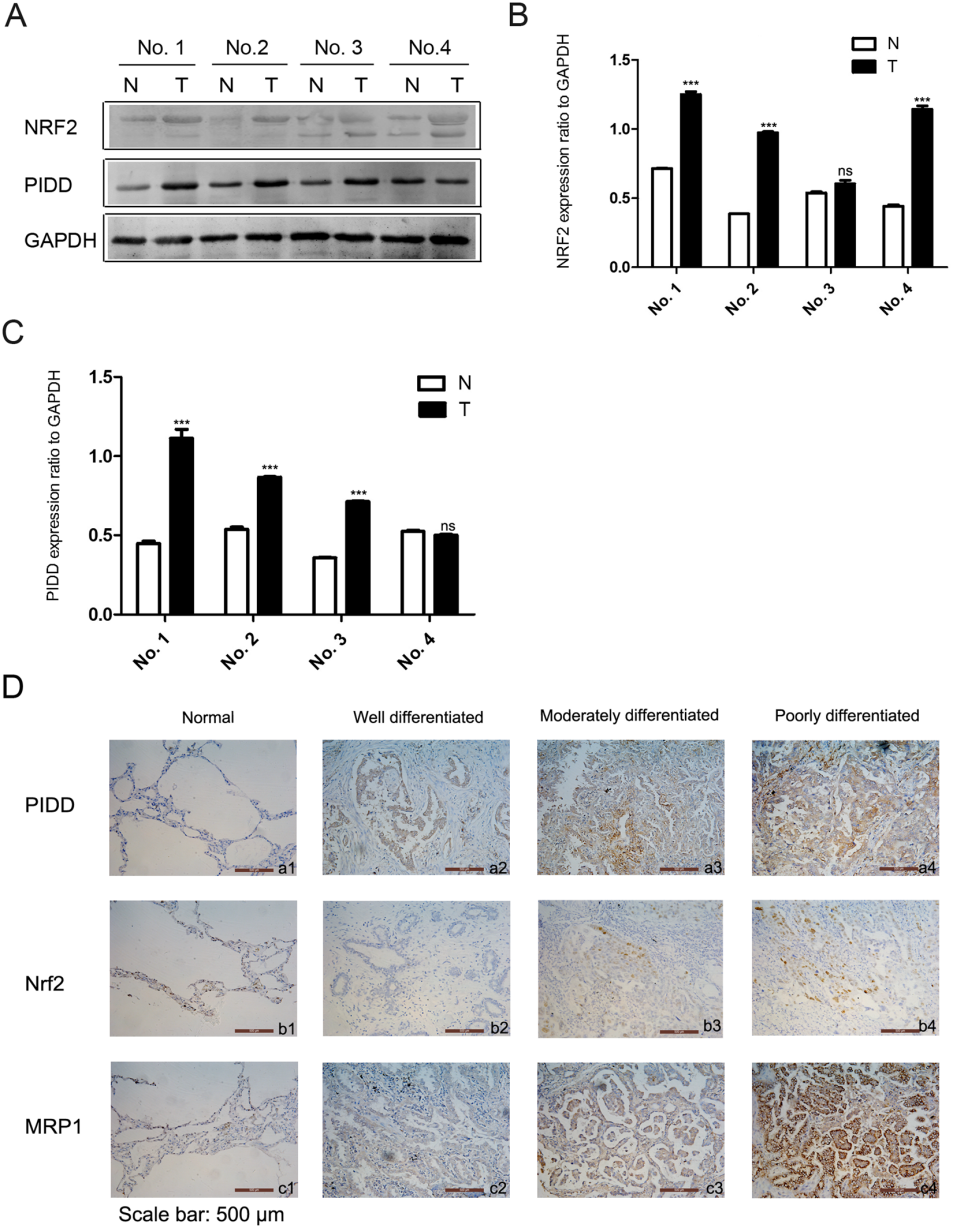
Expression of NRF2, PIDD and MRP1 in NSCLC patient tissues. (**A–C**) Four paired samples of NSCLC and non-tumorous adjacent tissues were homogenized and subjected to Western blot analysis to determine expression of NRF2 and PIDD. N: Non-tumorous adjacent tissues. T: Tumor tissues. (**A**) Representative western blot analysis. GAPDH was used as a loading control. The upper and lower panels were from the same gel. The gel was transferred to the same membrane, which was cut to probe with different antibodies. The entire image of each exposed membrane is shown. (**B**,**C**) Quantification of NRF2 and PIDD expression in matched normal and tumor NSCLC tissue by western blotting. Three independent western blotting experiments were performed from the same patient tissue lysates. The data are the mean ± SEM (Two tailed Student’s t-test; ***P < 0.001; ns, P > 0.05). (**D**) Immunohistochemical staining of PIDD (a1-a4), NRF2 (b1-b4), and the NRF2 target, MRP1 (c1-c4) in NSCLC specimens. Paraffin-embedded tissue sections were stained with antibodies against PIDD, NRF2, and MRP1, and counterstained with hematoxylin. Representative images from adjacent tissue normal (a1–c1,), and well differentiated (a2–c2), moderately differentiated (a3–c3), and poorly differentiated tumor tissue (a4–c4) are shown.

We next investigated PIDD and NRF2 expression in NSCLC patient tissue to obtain potential corroborating evidence for the clinical relevance of our findings. First, we compared PIDD and NRF2 expression in 4 pairs of matched NSCLC and adjacent normal tissues using Western blot analysis. NRF2 and PIDD levels were elevated in the tumor samples of 3 of the 4 cases, with 2 cases showing elevation in both PIDD and NRF2 in the tumor (Fig. 5A–C).

**Figure 6:**
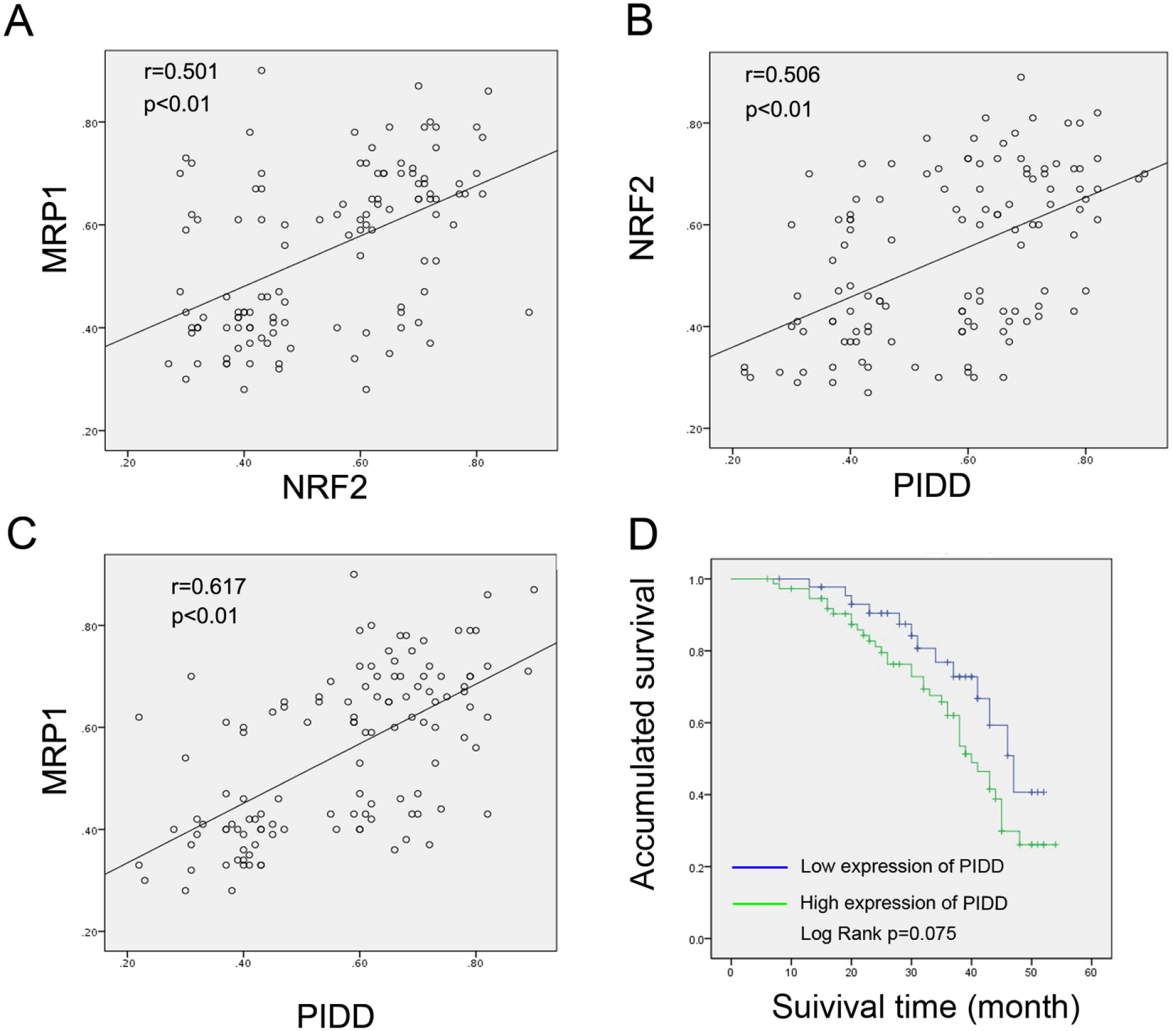
Correlations between NRF2, PIDD, and MRP1 expression and relationship of PIDD expression to survival in NSCLC patients. (**A–C**) Relationships between expression percentages of PIDD, NRF2, and MRP1, as assessed by IHC. Data were analyzed using the Software Package for Social Science (SPSS). Pearson correlation test (two-tailed) was used to analyze the relationship among the three, and p < 0.01 was taken as significant difference in all cases. (**A**) NRF2 and MRP1. (**B**) PIDD and NRF2. (**C**) PIDD and MRP1. (**D**) Kaplan-Meier survival analysis based on PIDD IHC expression status. A Log Rank (Mantel-Cox) test was performed to determine the experimental differences.

We then semi-quantitatively examined expression of PIDD, NRF2, and the NRF2 target gene, MRP1^31^, in 120 cases of treatment-naïve lung adenocarcinoma by immunohistochemistry (Supplemental Table S2, Fig. 5D). Using these data, we then evaluated the degree to which NRF2 might be active in the samples of this cohort by comparing NRF2 and MRP1 expression. The expression of these two proteins was significantly correlated, supporting NRF2 being active in many samples (Fig. 6A). We also found that PIDD expression was correlated with both NRF2 and MRP1, consistent with PIDD levels promoting NRF2 expression and activity (Fig. 6B,C).

Finally, we separated PIDD, NRF2, and MRP1 expression into low and high groups (see Methods) and re-evaluated their expression relative to each other, as well as to clinicopathologic variables, using the Pearson χ^2^ test (Table 1). Consistent with the Pearson correlation test analyses, significant positive correlations were found between the expression levels of PIDD and that of NRF2 and MRP1 (p = 0.037 and 0.038). There were also significant positive correlations between PIDD expression and that of tumor size (p = 0.007), lymph node status (p = 0.009), and clinical stage (p = 0.035). Overall, these data indicate that as compared with low PIDD expression, high PIDD levels are associated with larger tumor size, lymph node metastasis, and higher stage. Although not quite significant, Kaplan-Meier analysis indicated that there was a trend for high PIDD expression to also be associated with worse overall survival in this cohort that was treated with surgery alone (p = 0.075, Fig. 6D). These findings support PIDD regulating NRF2 activity in NSCLC and suggest that in addition to promoting chemoresistance, PIDD might also contribute to development of malignant properties in NSCLC.

**Table 1:** Clinicopathological features of lung adenocarcinoma in relation to PIDD expression.

Clinicopathological Features	Total	PIDD		p value
		low(N = 45)	high(N = 75)	
Gender				
Male	63(100%)	21(33.3%)	42(66.7%)	0.35
Female	57(100%)	24(42.1%)	33(57.9%)	
Age (years)				
<60	53(100%)	18(34%)	35(66%)	0.414
≥60	67(100%)	27(40.3%)	40(59.7%)	
Tumor size (cm)				
<3	52(100%)	27(51.9%)	25(48.1%)	0.007*
≥3	68(100%)	18(26.5%)	50(73.5%)	
Smoking status				
Yes	88(100%)	30(34.1%)	58(65.9%)	0.209
No	32(100%)	15(46.9%)	17(53.1%)	
Lymph node status				
0	64(100%)	31(48.4%)	33(51.6%)	0.009*
>0	56(100%)	14(25%)	42(75%)	
Clinical stage				
I	70(100%)	33(47.1%)	37(52.9%)	0.035*
II	32(100%)	8(25%)	24(75%)	
III	18(100%)	4(22.2%)	14(77.8%)	
NRF2 expression				
Low	56(100%)	27(48.2%)	29(51.8%)	0.037*
High	64(100%)	18(28.1%)	46(71.9%)	
MRP1 expression				
Low	62(100%)	29(46.8%)	33(53.2%)	0.038*
High	58(100%)	16(27.6%)	42(72.4%)	

Statistical analyses were performed by Pearson χ^2^ test.

*p < 0.05 was considered significant.

The details of how expression was classified into high and low groups are described in “Materials and methods”.

## Discussion

A wide body of work indicates that prolonged activation of the NRF2 transcription factor can promote progression of various cancers, including lung, breast, head and neck, ovarian, and endometrial carcinomas^32^. The poor prognosis of patients with tumors expressing high levels of NRF2 appears to relate to NRF2’s ability to both enhance cancer cell proliferation and promote chemo and radio-resistance^26,33,34^. In addition, NRF2 expression is induced during therapy, which further promotes development of drug resistance^35,36^. As such, NRF2 contributes to both intrinsic and acquired chemo-resistance^34,37^. Although NRF2 stabilization through genomic alterations in *NRF2*, *KEAP1*, and *CUL3* is well documented^8^, additional mechanisms that increase NRF2 activity have remained enigmatic and may play an equal or even greater role in cancer progression. Indeed, we found that although in the TCGA lung adenocarcinoma data, the cumulative alteration frequency of *NRF2*, *KEAP1*, and *CUL3* is 23% (Supplementary Fig. S1), 58% of patients within our lung adenocarcinoma cohort had high levels of NRF2 protein (Table 1).

One way through which NRF2 levels may be increased in the absence of genomic alterations in the three core genes is through an indirect mechanism involving competitive sequestration of KEAP1. p62, WTX, PALB2 and DPP3 have been reported to activate NRF2 by disrupting NRF2-KEAP1 binding^20,21,38^. However, the expression of these proteins in patient tumors and their potential contributions to tumor growth or chemoresistance are unknown. Here, we not only identified PIDD as a novel KEAP1-interactor, but also demonstrated its ability to promote NRF2 stabilization, which is accompanied by an increase in chemoresistance in NSCLC cells *in vitro* and *in vivo*.

PIDD was originally described as a primary p53 target gene that is induced upon DNA damage^39^. Despite initially being characterized for its role in pro-apoptotic pathways, PIDD was later shown to play a role in DNA-damage induced NF-kB activation, which putatively promotes cell survival and chemoresistance^40,41^. Indeed, later work, including in a KRAS-driven mouse lung cancer model and *KRAS* mutant human lung cancer cell lines, showed that PIDD overexpression can promote resistance to cisplatin^23,42^. Consistent with these findings, our functional studies using H1299 lung cancer cells also support PIDD expression promoting chemoresistance, and in cells that do not harbor *KRAS* mutations. However, whereas previous work in murine KRAS-driven tumors found some evidence that cisplatin induced changes in glutathione metabolism, a target pathway of NRF2^43^, those changes were not linked specifically to PIDD, and more evidence was presented that chemoresistance was primarily due to an increase in DNA repair mechanisms^23^. Our findings that high levels of PIDD prevent NRF2 ubiquitination and promote its stabilization, and are associated with increased NRF2 and target gene expression in NSCLC patient samples, suggest that PIDD-mediated NRF2 activation may have also been a major factor driving chemoresistance in the mouse lung tumors.

Our data thus, suggest a new way through which PIDD may affect tumor growth in response to DNA damaging chemotherapeutics. Previous work has described both pro and anti-apoptotic functions of PIDD that are mediated through distinct interactions involving proteins other than KEAP1^44^. The switch in the role of PIDD has also been linked to differential association with either PIDDosome complex containing Caspase-2 and RAIDD for cell death or NEMO (NF- kB)/RIP1 for cell survival^45^. In addition, PIDD also participates directly in DNA damage repair. PIDD binds to PCNA upon UV irradiation through its ZU-5 domains and stimulates PCNA monoubiquitination during translesion DNA synthesis^46^. Therefore, it is likely that multiple interactions between PIDD and other proteins may mediate its many roles in cellular responses to genotoxic stress.

Several inhibitors of NRF2 activity have been described. One group consists of non-specific natural products such as brusatol. Although brusatol sensitizes a broad spectrum of cancer cells to antitumor drugs and reduces cell growth^13,47^, its potent cytotoxic effects appear to act independently of NRF2 inhibition and are more consistent with the profile of a protein translation inhibitor^48^. Other non-specific inhibitory natural products include luteolin and alkaloid trigonelline^49,50^. By contrast a more specific NRF2 inhibitor, ML385, was recently identified through screening a drug library for compounds that inhibit NRF2-dependent transcriptional activity^51^. ML385 binds directly to NRF2 and enhances the cytotoxicity of platinum-based drugs on NSCLC cell lines. However, despite the promise of these anti-NRF2 therapies, the development of clinically useful drugs is still in its infancy.

Our finding that PIDD and NRF2 are widely co-expressed in clinical NSCLC support the PIDD-KEAP1 interaction as being prominent both in chemo-naïve and chemo-treated tumors. Thus, drugs that disrupt the PIDD-KEAP1 interaction may be especially effective in overcoming chemoresistance. However, given that PIDD can interact with different proteins to perform distinct functions, drugs should ideally be designed to interfere with the KEAP1 interaction, but potentially allow interaction with pro-apoptotic RAIDD. Indeed, some drugs are currently in clinical development that target specific protein-protein interactions^52^. For example, drugs are being developed to specifically interfere with human murine double minute 2 (MDM2) interaction withTP53. When MDM2 interacts with TP53, it promotes TP53 degradation, thereby antagonizing its tumor suppressor function^53^. Drugs such as NVP-CGM097 and NVP-HDM201 block the MDM2-TP53 interaction and are currently being tested in phase 1 clinical trials as potential anti-cancer therapies for tumors with wild-type *TP53*^54^.

In conclusion, our work supports continued study of non-genomic mechanisms of NRF2 activation and further characterization of PIDD and KEAP1 interactions with their distinct binding partners.

## Materials and Methods

### Mass spectrometry

One round of discovery immumoprecipitation-mass spectrometry (IP-MS) analysis was performed using H1299 NSCLC cells. Cells were lysed in immunoprecipitation buffer (25 mM Tris-HCl (pH 7.5), 150 mM NaCl, 1 mM EDTA, and 1% NP-40, pH 7.8) and precleared with protein G sepharose (Sigma) for 2 h. Then, 100 mg of protein lysate was immunoprecipitated overnight at 4 °C with 5 μg of anti-KEAP1 antibody (H-190; SC 33569, Santa Cruz Briotechnology, Inc.), or rabbit normal immunoglobulin G (IgG) (Bioworld Technology, MN). Immune complexes were recovered with protein G sepharose for 2 h. After three washes in lysis buffer, the samples were run on a 10% SDS-PAGE gel and stained with Coomassie brilliant blue. The gel lanes were cut out and proteins extracted and subjected to mass spectrometry analysis as previously described^55^.

### Tissue specimens

Permission for the use of the human fresh tissue samples, tissue sections, and associated patient clinical data for this project was granted by the Ethics Committee of the Affiliated Hospital of Nantong University, Jiangsu Province, China. Written informed consent was obtained from all the patients, and patient specimens were collected in accordance with the Declaration of Helsinki 2013. For Western blot analyses, a total of four paired fresh NSCLC tumorous and adjacent non-tumorous lung tissues were used. These samples were harvested immediately after surgical resection and stored at −80 °C. For immunohistochemistry analyses, 120 lung adenocarcinoma specimens collected by informed consent from 2008 to 2013, were obtained from the Department of Pathology, Affiliated Hospital of Nantong University. None of the patients had received pre-surgical therapies such as immunotherapy, radiation, or chemotherapy. The paraffin sections were stained with hematoxylin and eosin (H&E). Demographic information and clinical and pathological data of these 120 patients are shown in Supplementary Table S3.

### Immunohistochemical staining

For immunohistochemical staining, deparaffinized sections were processed for antigen retrieval by heating in sodium citrate buffer (pH 6.0). The following panel of primary antibodies was used: (1) NRF2 (1:100, ab76026, Abcam, Inc.), (2) MRP1 (1:50, ab24102, Abcam, Inc.), and (3) PIDD (1:100, sc-32161, Santa Cruz Briotechnology, Inc.). The sections were incubated with primary antibody for 1 h at 37 °C and 4 °C overnight. After washing with phosphate-buffered saline (PBS), tissues were incubated with a horseradish peroxidase-conjugated anti-rabbit or anti-mouse Ig polymer as a second antibody (Dako, Hamburg, Germany) for 20 min at room temperature, according to the manufacturer’s instructions. Finally, the peroxidase reaction was visualized by incubating the sections with DAB (0.1% phosphate buffer solution, 0.02% diaminobenzidine tetrahydrochloride, and 3% H_2_O_2_). After rinses in water, the sections were counterstained with hematoxylin and then dehydrated and mounted in resin mount. Negative control slides were processed in parallel using a non-specific immunoglobulin IgG (Sigma Chemical Co, St. Louis, MO, USA) at the same concentration as the primary antibody.

### Immunohistochemical analysis

All samples were evaluated and independently scored by 2 pathologists using a prespecified protocol. Five high-power fields in each section were selected randomly, and at least 300 cells were counted per field. Each slide was evaluated using a semiquantitative scoring system for both the intensity of the stain and the percentage of positive malignant cells. The intensity of staining was coded as follows: 1 (negatively or poorly stained), 2 (moderately stained), and 3 (strongly stained). The percentage of staining was scored as: 1 (<50%), 2 (50–75%), and 3 (>75%). The two scores were then multiplied and samples classified into two groups: high expression (score ≥4.5) and low expression (score <4.5)^56,57^ (Supplementary Table S2).

### Cell lines, cell culture, cell transfections and cell viability assay

The human NSCLC cell line H1299 was purchased from ATCC and was cultured in RPMI-1640 medium supplemented with 10% fetal bovine serum (FBS), and maintained in a 5% CO_2_ humidified atmosphere at 37 °C. The HEK293T cell line was purchased from ATCC and cultured in Dulbecco’s modified Eagle’s medium (DMEM) supplemented with 10% FBS.

Cells were transfected at 70–90% confluence, utilizing 2 µl of Lipofectamine (Invitrogen)/µg of DNA. The KEAP1 expression plasmid was a gift from Dr. Qing Zhong (Addgene plasmid #28023), which tags KEAP1 with a 3X FLAG epitope at the C terminus. The PIDD expression plasmid was obtained from GENEPPL (http://www.geneppl.com/#PPL00209-2a), which tags PIDD with an HA epitope at the N-terminus. Lipofectamine and DNA were incubated in serum-free DMEM for 5 min in separate tubes. After incubation, the contents of two tubes were combined, incubated for an additional 15 min, and applied onto cells.

Cell viability was measured using the 3-(4,5-dimethylthiazol-2-yl)-2,5-diphenyl tetrazolium bromide (MTT) Cell Proliferation Kit (Sigma-Aldrich Com).

### Western blot analysis and immunoprecipitation

The tissues and harvested cells were promptly homogenized in lysis buffer (50 mM Tris–HCl, pH 7.4, 150 mM NaCl, 1% NP-40, 5 mM EDTA, and a protease inhibitor cock-tail (Roche Diagnostics, Basel, Switzerland)), and then centrifuged to collect the supernatant. Protein concentrations were measured with a Bio-Rad protein assay (Bio-Rad, Hercules, CA, USA). Next, the same total protein was separated by SDS–PAGE and transferred to a polyvinylidene difluoride (PVDF) membrane (Immobilon; Millipore). The membranes were first blocked with 5% non-fat milk in Tris-buffered saline with Tween 20 (TBST) (150 mM NaCl, 20 mM Tris, 0.05% Tween 20), washed with TBST for three times after 2 h blocking at room temperature. The membrane was then cut according to the position of corresponding protein, and probed overnight with each primary antibody. After three washes, the membranes were incubated with horseradish peroxidase-conjugated secondary antibodies (Pierce Biotechnology) for 2 h at room temperature, and proteins detected by ECL (enhanced chemiluminescence) (Pierce, Rockford, IL). Alternatively, secondary antibody incubation was performed using fluorescent secondary antibodies (LI-COR Biosciences, Lincoln, NE), and protein bands were visualized using an Odyssey system (LI-COR Biosciences).

For immunoprecipitation assays, H1299 cells were grown to confluence in 10-cm dishes and washed with cold phosphate-buffered saline (PBS). For HEK293T cells, they were transiently transfected with 10 µg of the expression plasmids by using Lipofectamine (Invitrogen) according to the manufacturer’s instructions. At 48 hours after transfection, the growth medium was aspirated and cells were washed three times with cold PBS. The supernatants of these cell lysates were immune-precipitated with the primary antibodies against PIDD (sc-32161, Santa Cruz Briotechnology, Inc.), KEAP1 (H-190; SC 33569, Santa Cruz Briotechnology, Inc.), HA (ab9110, Abcam), FLAG (ab1240, Abcam), or control IgG (Santa Cruz Biotechnology) conjugated to protein G-Sepharose beads. Ten micrograms of antibody were added to 500 micrograms of cell lysate, followed by a 2 h incubation with gentle rotation at 4 °C. The precipitates were washed three times with PBS and collected for Western blot analysis.

### Construction of PIDD overexpressing H1299 cells by lentiviral infection

The PIDD lentiviral overexpression plasmid was constructed and purchased from Genechem (Genechem Co., Ltd, Shanghai, China). For amplification of PIDD by polymerase chain reaction (PCR), a pair of primers were designed and synthesized as follows: Forward primer: 5′-GAGGATCCCCGGGTACCGGTCGCCACCATGGCTGCAACGGTGGAGGGGCCAGAG-3′; Reverse primer: 5′-TCCTTGTAGTCCATACCGGCCTGGGCAGGCTCTGGGGGCTGTG-3′. The resulting PIDD cDNA was inserted into the lentiviral vector GV358 (sequence elements: Ubi-MCS-3XFLAG-SV40-GFP-IRES-puromycin; Shanghai Genechem Company, China) to create the lentiviral vector, LV-PIDD. Sequencing was performed to verify the correctness of the sequence. For lentivirus packaging, 293 T cells were cotransfected with the recombinant PIDD expression vector (LV-PIDD), as well as the packaging plasmids, Helper 1.0 and Helper 2.0 (Genechem Company, Shanghai, China) to generate the target lentivirus with an infectious viral titer of 2 × 10^8^ TU/ml, which was measured using a fluorescence assay method. The harvested lentiviruses were concentrated, purified and stored at −80 °C.

For PIDD overexpression studies, H1299 cells were exposed to either LV-PIDD lentiviral particles in the presence of polybrene (8 μg/ml, Sigma-Aldrich) or mock conditions (control), in which cells were exposed to media containing polybrene, but no virus. After 8 h, the medium was replaced. Stable PIDD overexpressing cells were selected by treatment with puromycin (Sigma-Aldrich), while mock-infected cells were grown in the absence of puromycin.

### NRF2 knockdown in MGH7 and H1299-LV-PIDD cells

NRF2 shRNA lentiviral constructs in the pLKO.1 backbone were purchased from Sigma-Aldrich, St. Louis, USA. The NRF2 targeting sequences included CCGGGCACCTTATATCTCGAAGTTTCTCGAGAAACTTCGAGATATAAGGTGCTTTTT for shRNA1 and CCGGGCTCCTACTGTGATGTGAAATCTCGAGATTTCACATCACAGTAGGAGCTTTTT for shRNA2. The NRF2-shRNA plasmids or non-targetting lacZ shRNA negative control plasmid, along with standard helper packaging plasmids, were transfected into 293T cells using FuGENE 6 (Promega, Madison, WI), following the manufacturer’s instructions, to make virus. The viral supernatant was then applied to MGH7 and H1299-LV-PIDD cells, and after 48 hours, the cells were subjected to puromycin selection (Sigma-Aldrich, St. Louis, USA, 3 ug/ml) for 72 hours.

### Tumor formation in nude mice

BALB/c athymic nude male mice (4 to 6 weeks old) were purchased from Shanghai SLAC Animal Center and raised in a specific pathogen-free environment in Laboratory Animal Centre of Nantong University. Animal care and all experiment procedures were reviewed and approved by Jiangsu Institutes of Health Guide for the Care and Use of Laboratory Animals. LV-PIDD and LV-Mock infected H1299 cells (1 × 10^7^ cells) were collected, resuspended in 200 µl PBS, and injected subcutaneously into each mouse (5 mice per each arm) to create the two arms of the study. At 10 days of tumor formation, all mice (both arms) received an intraperitoneal injection of cisplatin (7.5 mg/kg). Tumor growth was monitored until the day that mice were sacrificed. The tumor volume was measured every 5 days with a vernier caliper and calculated with the following formula: volume (mm^3^) = length × width × height × 0.5^58^. Animals were sacrificed 30 days after injection.

### Statistical analysis

A Pearson correlation test was performed to compare the expression levels of PIDD, NRF2 and MRP1 by twos. The expression of PIDD and its relationship to clinicopathological features was analyzed using Pearson χ^2^ test. Kaplan–Meier survival plots were used to analyze survival data with a log-rank test used to determine significance. For all statistical analyses, the level of significance was set at p < 0.05. Statistical analysis was performed with Graph Pad Prism 5 and the Software Package for Social Science (SPSS) 19.0 statistical software (SPSS, Inc. Chicago, IL, USA). All values were expressed as mean ± SEM.

## Supplementary information

Dataset 1

Dataset 2

Dataset 3

## Data Availability

The datasets that were generated and/or analyzed during the current study are available from the corresponding author upon request.
