# Supplementary material for: PIDD interaction with KEAP1 as a new mutation-independent mechanism to promote NRF2 stabilization and chemoresistance in NSCLC
Source: Sci Rep. 2019 Aug 27;9:12437. doi: 10.1038/s41598-019-48763-4 (PMC6712044; doi:10.1038/s41598-019-48763-4)
Supplement: Supplementary file 1 — Dataset 1 [file 41598_2019_48763_MOESM1_ESM.pdf]

PIDD interaction with KEAP1 as a new mutation-independent mechanism to promote NRF2 stabilization and chemoresistance in NSCLC

Lili Ji, Rui Zhang, Jie Chen, Qun Xue, Nadeem Moghal, Ming-Sound Tsao

Supplementary Figure S1. NRF2 regulatory pathway alterations in NSCLC and other cancers
The cBioportal database at www.cBioportal.org was used to determine genetic alteration frequencies (amplifications, deletions, and mutations) across different cancer types, using the TCGA Provisional data set with n=7767 sequenced patients. (A) Cumulative alteration frequencies of all three genes including *NRF2*, *KEAP1*, and *CUL3* in 31 surveyed malignancies. (B) The detailed alteration frequencies for NRF2 in 31 surveyed malignancies. (C) The detailed alteration frequencies for KEAP1 in 31 surveyed malignancies. (D) The detailed alteration frequencies for Cul3 in 31 surveyed malignancies.

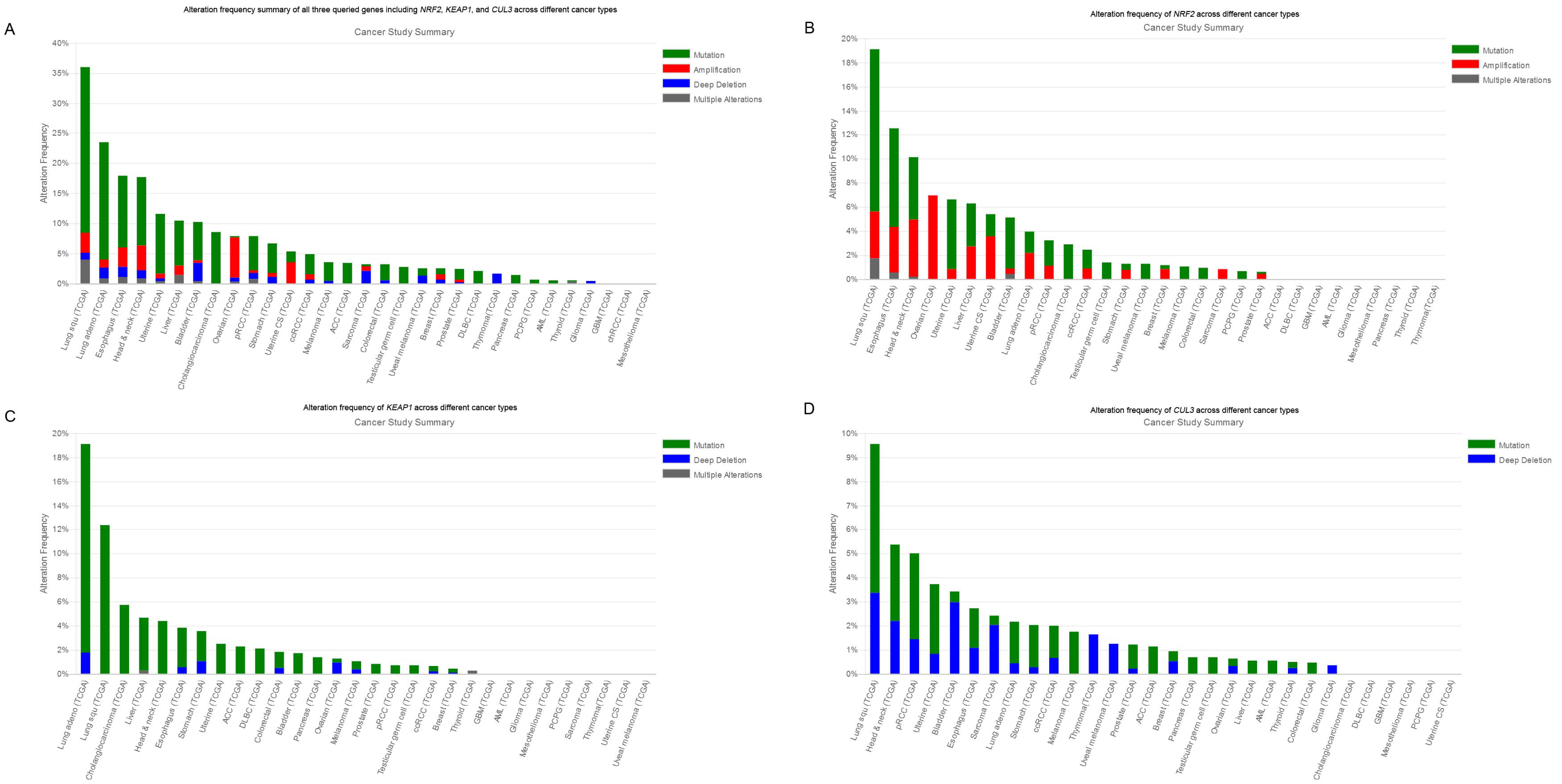
A
Alteration frequency summary of all three queried genes including *NRF2*, *KEAP1*, and *CUL3* across different cancer types
Cancer Study Summary
Alteration Frequency
Mutation
Amplification
Deep Deletion
Multiple Alterations
Lung squ (TCGA)
Lung adeno (TCGA)
Esophagus (TCGA)
Head & neck (TCGA)
Uterine (TCGA)
Liver (TCGA)
Bladder (TCGA)
Cholangiocarcinoma (TCGA)
Ovarian (TCGA)
pRCC (TCGA)
Stomach (TCGA)
Uterine CS (TCGA)
ccRCC (TCGA)
Melanoma (TCGA)
ACC (TCGA)
Sarcoma (TCGA)
Colorectal (TCGA)
Testicular germ cell (TCGA)
Uveal melanoma (TCGA)
Breast (TCGA)
Prostate (TCGA)
DLBC (TCGA)
Thymoma(TCGA)
Pancreas (TCGA)
PCPG (TCGA)
AML (TCGA)
Thyroid (TCGA)
Glioma (TCGA)
GBM (TCGA)
chRCC (TCGA)
Mesothelioma (TCGA)
B
Alteration frequency of *NRF2* across different cancer types
Cancer Study Summary
Alteration Frequency
Mutation
Amplification
Multiple Alterations
C
Alteration frequency of *KEAP1* across different cancer types
Cancer Study Summary
Alteration Frequency
Mutation
Deep Deletion
Multiple Alterations
D
Alteration frequency of *CUL3* across different cancer types
Cancer Study Summary
Alteration Frequency
Mutation
Deep Deletion

Supplementary Table S3. Clinicopathological characteristics of patient samples

Factor	n	Mean ± SE
Gender		
Male	63	
Female	57	
Age (years)		
<60	53	52.83 ± 0.76
≥60	67	66.87 ± 2.10
Tumor size (cm)		
<3	52	
⩾3	68	
Smoking status		
Yes	88	
No	32	
Lymph node status		
0	64	
>0	56	
Clinical stage		
I	70	
II	32	
III	18	
